# Maternal COVID-19 causing intrauterine foetal demise with microthrombotic placental insufficiency: a case report

**DOI:** 10.1186/s12884-023-05942-6

**Published:** 2023-09-09

**Authors:** Olivia Nonn, Lilli Bonstingl, Katja Sallinger, Lena Neuper, Julia Fuchs, Martin Gauster, Berthold Huppertz, Dagmar Brislinger, Amin El-Heliebi, Herbert Fluhr, Eva Kampelmühler, Philipp Klaritsch

**Affiliations:** 1 Charité – Universitätsmedizin Berlin, corporate member of Freie Universität Berlin and Humboldt- Universität zu Berlin, Berlin, Germany; 2 Experimental and Clinical Research Centre, Max Delbrück Center for Molecular Medicine in the Helmholtz Association and Charité Universitätsmedizin Berlin, Berlin, Germany; 3 Max Delbrück Center for Molecular Medicine in the Helmholtz Association (MDC), Berlin, Germany; 4 Division of Cell Biology, Histology and Embryology, Gottfried Schatz Research Center, Medical University of Graz, Graz, Austria; 5 Center for Biomarker Research in Medicine (CBmed), Graz, Austria; 6 Division of Medical Physics and Biophysics, Gottfried Schatz Research Center, Medical University of Graz, Graz, Austria; 7 BioTechMed, Graz, Austria; 8 Department of Obstetrics and Gynaecology, Medical University of Graz, Graz, Austria; 9 Diagnostic and Research Institute of Pathology, Diagnostic and Research Center for Molecular Biomedicine, Medical University of Graz, Graz, Austria; 10 Research Unit for Fetal Medicine, Department of Obstetrics and Gynaecology, Medical University of Graz, Graz, Austria

**Keywords:** SARS–CoV-2-Infection, COVID-19, Vertical transmission, Intrauterine foetal death, *In situ* detection, Perivillous fibrin deposition and chronic placentitis, Pregnancy, Microthrombosis, Case report

## Abstract

**Background:**

Pregnant women have an increased risk of getting infected with SARS-CoV-2 and are more prone to severe illness. Data on foetal demise in affected pregnancies and its underlying aetiology is scarce and pathomechanisms remain largely unclear.

**Case:**

Herein we present the case of a pregnant woman with COVID-19 and intrauterine foetal demise. She had no previous obstetric or gynaecological history, and presented with mild symptoms at 34 + 3 weeks and no signs of foetal distress. At 35 + 6 weeks intrauterine foetal death was diagnosed. In the placental histopathology evaluation, we found inter- and perivillous fibrin depositions including viral particles in areas of degraded placental anatomy without presence of viral entry receptors and SARS-CoV-2 infection of the placenta.

**Conclusion:**

This case demonstrates that maternal SARS-CoV-2 infection in the third trimester may lead to an unfavourable outcome for the foetus due to placental fibrin deposition in maternal COVID-19 disease possibly via a thrombogenic microenvironment, even when the foetus itself is not infected.

**Supplementary Information:**

The online version contains supplementary material available at 10.1186/s12884-023-05942-6.

## Novelty

Symptomatic COVID-19 of the mother may cause microthrombotic events in the placenta.

These microthrombotic events may lead to placental insufficiency and reduced growth velocity.

Further systematic clinical studies investigating the thrombogenic effect of a SARS-CoV-2 infection in pregnancy are warranted.

Although a single case report, the findings support previous concerns about placental insufficiency after SARS-CoV-2 infection.

This report adds to existing literature and could help support recommendations for additional antenatal testing in similar settings.

**Supplementary Information:**

The online version contains supplementary material available at 10.1186/s12884-023-05942-6.

## Introduction

While pregnant women have double the risk of getting infected with SARS-CoV-2 [1], they are also more prone to severe illness, requiring intensive care admission and invasive ventilation more often [2–4]. Emerging evidence from a multinational meta-analysis suggests that rates of stillbirth have increased during the pandemic in general [5], but studies about the real risk of miscarriage in women with SARS-CoV-2 infection are contradictory [6–8]. Recent reports suggested placental infection with SARS-CoV-2 as a confounding factor for foetal demise in the second trimester [9].

Recent studies reported histopathological evidence of perivillous fibrin depositions in placentas from mothers with COVID-19 [10, 11]. Others found in a largely asymptomatic population, that SARS-CoV-2 infection in pregnancy is primarily associated with maternal inflammatory responses in the circulation and at the maternal–foetal interface, suggesting that acute foetal infection did not occur [12]. Another multicentre study detected an increased rate of preterm birth [13]. However, data on foetal demise in affected pregnancies and its underlying aetiology is still scarce and the pathomechanisms remain largely unclear.

In our report, we present a case of foetal demise in a SARS-CoV-2 infected gravida with extensive diagnostic workup.

## Results

This case demonstrates how maternal SARS-CoV-2 infection in the third trimester may lead to an unfavourable outcome for the foetus due to placental involvement in maternal COVID-19 disease, even when the foetus itself is not infected. Absence of viral entry receptors and SARS-CoV-2 infection of the placenta in areas of trophoblast necrosis was detected with highly specific probes. We speculate that placental inflammation due to maternal SARS-CoV-2 infection presents with massive intervillous and perivillous fibrin deposition and scant chronic placentitis, finally leading to placental insufficiency and foetal death.

### Clinical investigation

A 25-year-old primigravida presented at 34 + 3 weeks of gestation at the outpatient clinic of a local hospital with fever (39.2 °C), cough, fatigue, and abdominal pain. Pregnancy had been uneventful so far as well as the last regular check with her gynaecologist two days before. At admission, SARS-CoV-2 infection was documented via quantitative real-time-PCR (RT-qPCR) with a cycle threshold (Ct) value of 16.3 (Table 1).

**Table 1:** Maternal and foetal/neonatal clinical examination

**Visit 1**	***at 34 + 3 weeks***	
	**Maternal parameters**	
	*Temperature*	39.2 °C
	*Virology*	SARS-CoV-2-PCR positive
		RT-qPCR for viral membrane protein E-gene and viral nucleoprotein N-gene: SARS-CoV-2-E Ct-Value 16.3
		SARS-CoV-2-N2 Ct-Value 18.2
	**Foetal parameters**	
	*Estimated foetal weight*	2,380 g
		(38th centile)
	*Sonography*	normal amniotic fluid volume
		normal umbilical artery Doppler flow (0.79 PI)
		lively foetal movements
**Visit 2**	***at 35 + 6 weeks***	
	**Maternal parameters**	
	*Temperature*	37.7 °C
	*CBC*	haemoglobin 13.1 g/dl
		leukocytes 11.13 × 10^9^/l
		neutrophils 85.0% (9.46 × 10^9^/l)
		lymphocytes 8.70% (0.79 × 10^9^/l)
		platelets 258 × 10^9^/l
	*Clin. Chem.*	CRP 10 mg/l
		GGT 59.0 U/l
		LDH 312 U/l
	*Virology*	RT-qPCR for viral membrane protein E-gene and viral nucleoprotein N-gene: SARS-CoV-2-E Ct-Value 37.3
		SARS-CoV-2-N2 Ct-Value 35.2
	**Foetal/neonatal parameters**	
	*Sonography*	intrauterine foetal death
		no signs of placental abruption or preterm rupture of membranes
	*Autopsy*	2,176 g
		47 cm
		no structural malformations
		no dysmorphism
		signs of foetal distress (meconium aspiration)
		moderately autolysed viscera without inflammation
	*Virology*	RT-qPCR for viral membrane protein E-gene
		RT-qPCR for viral nucleoprotein N-gene negative in:
		• lungs • liver • brain
	**Placental parameters**	
	*Gross pathology*	310 g (10th centile)
		13.5 × 12 cm
		increased consistency
		generalised fibrin deposition
	*Virology*	viral membrane protein E-gene positive
		viral nucleoprotein N-gene positive

Standard antenatal care in Austria includes sonographic foetal evaluation, which at this point revealed appropriate biometric data with an estimated foetal weight of 2,380 g (38th centile), normal amniotic fluid volume, normal umbilical artery Doppler flow (pulsatility index 0.79), normal foetal cardiac activity, and normal foetal movements. There were no signs for premature rupture of membranes or urinary infection or any other obstetric pathology. The patient was counselled about home quarantine, received a prescription for Paracetamol 500 mg orally as well as daily subcutaneous Enoxaparin 4000 IU and was discharged from hospital. As the case has occurred before the approval of Nirmatrelvir/Ritonavir by the European Medicines Agency, no antiviral medication was considered.

Ten days later, at 35 + 6 weeks of gestation, she returned to hospital due to absent foetal movements for two days. Sonography confirmed intrauterine foetal death without signs of placental abruption or preterm rupture of membranes. The patient was asymptomatic regarding COVID-19, but still positive for SARS-CoV-2 with a Ct value of 37.3. Temperature was 37.7 °C. Laboratory findings included a complete blood count (CBC) showing haemoglobin 13.1 g/dl, leukocytes 11.13 × 10^9^/l, neutrophils 85.0% (9.46 × 10^9^/l), lymphocytes 8.70% (0.79 × 10^9^/l), platelets 258 × 10^9^/l, and clinical chemistry for CRP 10 mg/l, GGT 59.0 U/l and LDH 312 U/l.

She received misoprostol and oxytocin for induction of labour and delivered a stillborn girl (2,176 g) nine hours later. A single loop of umbilical cord lay around the infant’s neck and amniotic fluid was meconium-stained. The further course was uneventful, and the patient was discharged from hospital two days later.

The stillborn girl and the placenta were transferred to the Diagnostic and Research Institute of Pathology at the Medical University of Graz for further examination.

### Foetal autopsy report

The female foetus’ weight at autopsy of 2,176 g and its length of 47 cm correspond to the 35th week of gestation. No structural malformations or dysmorphisms were observed. Deeply aspirated squames and meconium raised the suspicion of foetal distress. The viscera were moderately autolysed, without any sings for inflammation. The time of death was estimated to be approximately one week before stillbirth.

### Histopathological placental evaluation

**Fig. 1:**
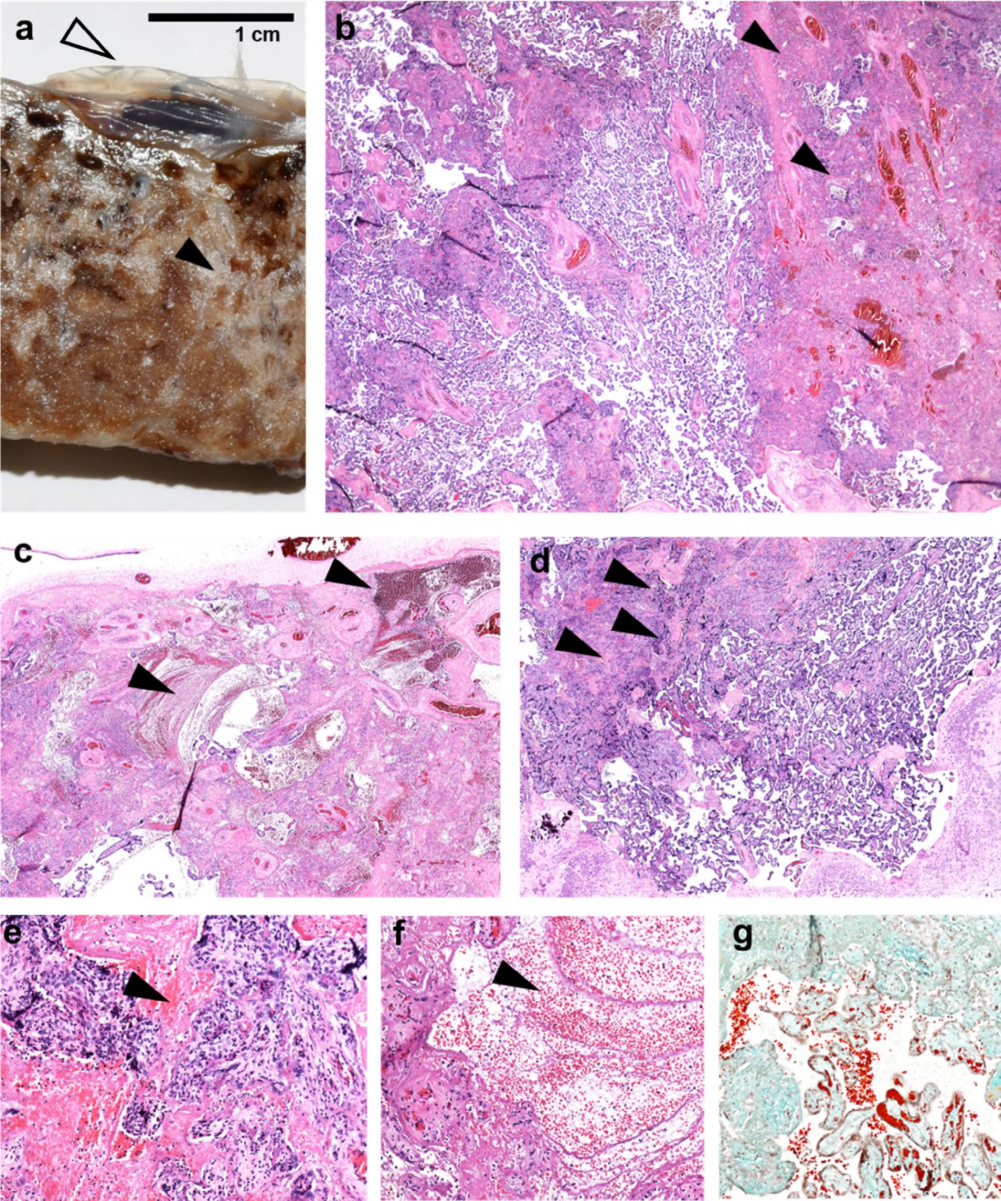
Histopathological examination of the placenta confirming generalised fibrin deposition and microthrombotic deposition. **(a)** generalised fibrous changes throughout the whole cross-section of parenchyma from the foetal side (empty arrowhead) consistently towards the basal plate at the maternal side, with visible recent and consolidated fibrin deposition throughout (black arrowheads in a) consistent with a protracted subacute course of perivillous microthrombosis. **(b)** fibrin deposition (black arrowheads) visible (H&E staining). **(c)** Microfibrinous areas in subchorionic layers (arrowheads). **(d)** Microfibrinous areas in basal layers (arrowheads). **(e,f)** intervillous space filled with thrombi and fibrinoid instead of maternal blood (arrowheads). **(g)** subacute fibrin deposition, featuring recent fibrin deposition in orange and older fibrin deposition in green (fibrin staining - M/G Masson/Goldner); (b,c,d in 20x magnification, e,f,g in 100x magnification)

The trimmed placenta measured 13.5 × 12 cm and weighed only 310 g which is below the 3rd centile at the 35th week of gestation [14]. The umbilical cord presented with livid changes (24 to 1 cm from insertion site). The placental consistency was remarkably increased. Incision exposed abundant fibrin deposits throughout the whole parenchyma, blocking approximately two thirds of the maternal-foetal-interface (Fig. 1a). The alterations resembled those found in preeclampsia but without maternal infarcts or haemorrhages. Visible recent and consolidated fibrin deposition (Fig. 1b,c,d,g) were found throughout, consistent with a protracted subacute course of perivillous microthrombosis (Fig. 1e,f). Taken together, the findings suggest a placental growth arrest at the time of symptom-onset, most likely due to growth restriction with perivillous fibrin depositions. Extrapolating the placental pathology measurements, the placental size and weight is consistent with standard measurements found at the 34th week of gestation.

Brightfield microscopy confirmed extensive perivillous fibrin deposition (Fig. 1), mostly associated with necrosis of villous trophoblast. Cytotrophoblast and syncytiotrophoblast nuclei were lacking the nuclear staining in areas of fibrin deposits, whereas signs of foetal circulation could still be detected in underlying villous stroma. Some lymphohistiocytic infiltrates were present in villous stroma, without polymorphonuclear leukocytes. Meconium-laden macrophages were present in foetal membranes; the latter as well as the umbilical cord were not inflamed.

### Molecular analyses

There were no signs for foetal viral infection. SARS-CoV-2 was not detectable by RT-qPCR for SARS-CoV-2 viral RNA amplification (human *GAPDH* gene and viral E gene, N gene) in foetal lungs, liver and brain tissue.

RT-qPCR for viral membrane protein E, viral nucleoprotein N, and human housekeeping gene *GAPDH* demonstrated positive results for viral sequences of placental samples containing maternal blood (Table 1).

**Fig. 2:**
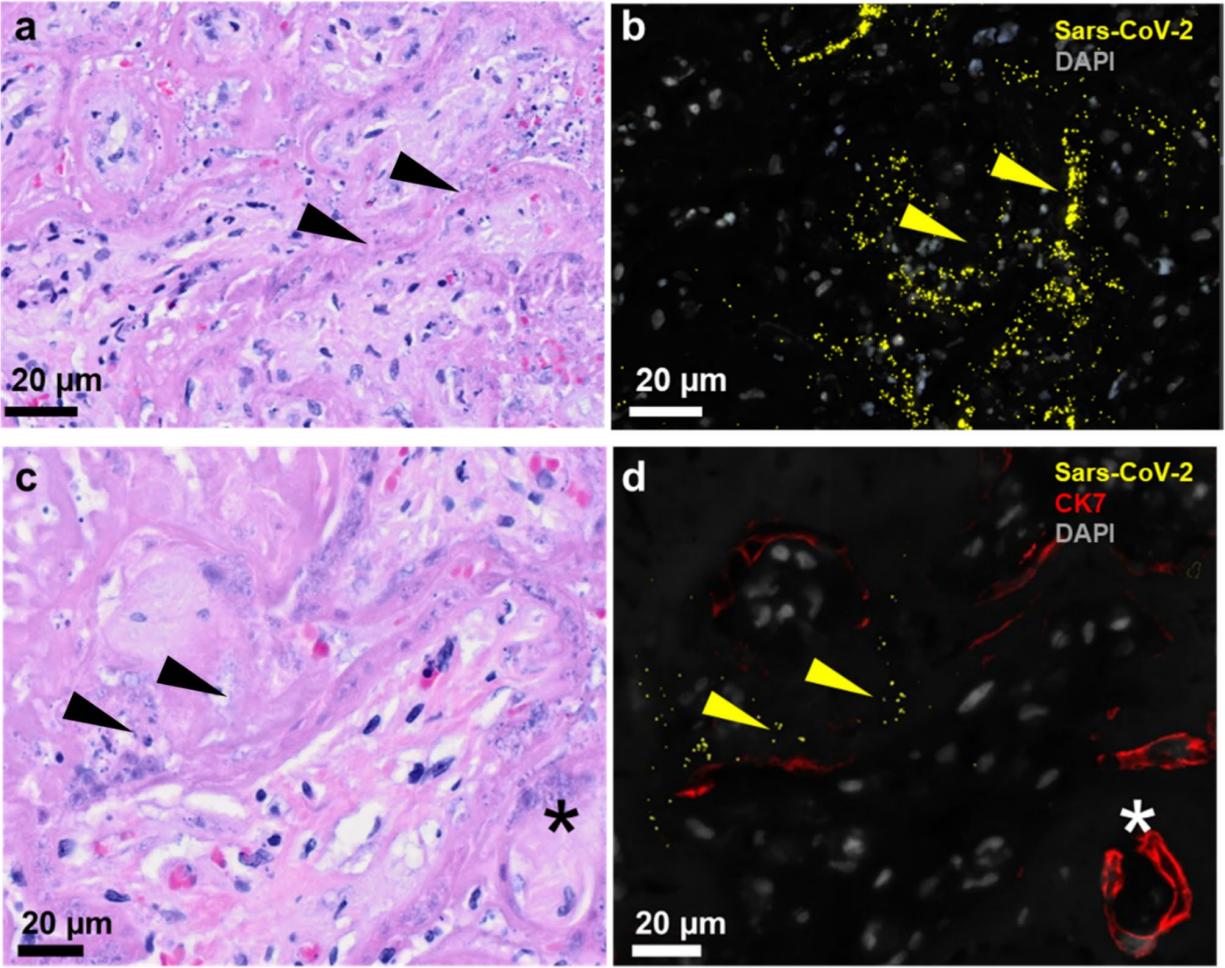
SARS-CoV-2 mRNA detected at sites with trophoblast lacking cytokeratin 7 staining. **(a)** H&E-staining showing the structure in the area of the consecutive section where **(b)** SARS-CoV-2-RNA (yellow arrowheads) is detected by padlock probe hybridisation. **(c)** Fibrin deposition throughout the intervillous space, the same area in a consecutive slide showing **(d)** SARS-CoV-2-RNA detection (yellow arrowheads) combined with trophoblast marker cytokeratin 7 indicating an almost intact trophoblast layer of a villous cross-section (asterisk *). DAPI recoloured in grey

Expanded investigation with mRNA in situ hybridisation offered further spatial insight into the viral infection of the placenta (Fig. 2a, b). In situ hybridisation was combined with immunofluorescence for the pan-trophoblast marker cytokeratin 7. We detected viral mRNA in regions with absent cytokeratin-7 staining (Fig. 2d). This is concordant with areas of perivillous fibrin deposition attached to sites of trophoblast necrosis as seen in the serial sections stained with H&E (Fig. 2a, c).

### Virus entry assessment

**Fig. 3:**
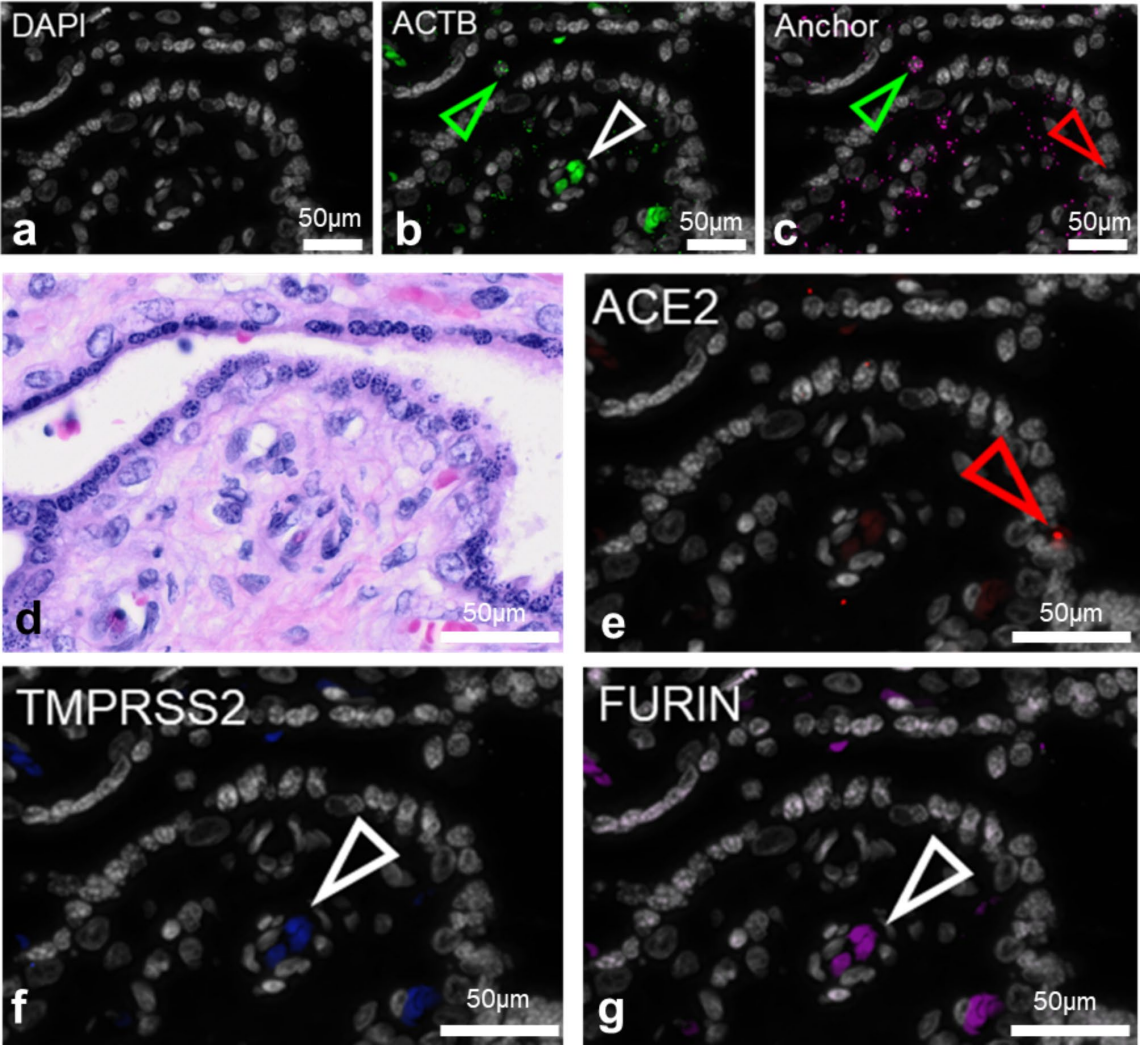
Viral entry receptors *ACE2, FURIN, TMPRSS2* are not expressed in the placenta. DAPI recoloured in grey **(a)**. RNA is detected by padlock probe hybridisation using one specific hybridisation probe with ACTB **(b)** as positive control, one common probe (anchor; **c**) and specific targets *ACE2*, *TMPRSS2*, and *FURIN* **(e-g)**. H&E-staining showing the structure in the area of the consecutive section **(d)**. Specific signals are double-positive for the anchor and one specific stain (indicated by green arrowheads in positive control ACTB, **b**) and overlayed with DAPI (**a**). Unspecific false positive signals are not colocalised with anchor signals and/or visible in several channels (red arrow showing unspecific signal in **c** and **e**), while white arrowheads in **b, f, g** show autofluorescent erythrocytes detectable in several channels but the anchor probe image, **c**). Scale bars show 50 μm

Syncytiotrophoblast did not express any of the known viral entry receptors, i.e. angiotensin converting enzyme *ACE2*, serin protease *TMPRSS2* and paired basic amino acid cleaving enzyme *FURIN* (Fig. 3). These receptors were neither expressed on the surface of viable villi nor in those under massive perivillous fibrin deposition. In contrast, numerous viral signals could be found within the perivillous fibrin deposition (Fig. 2).

## Discussion

We present a case of maternal SARS-CoV-2 infection during the delta-variant spread with subsequent fetal death and microthrombotic depositions in the placenta. In a previously reported case with foetal death associated with mild COVID-19 in a 34-week-pregnant woman, a single alteration of a heterozygous pattern for Leiden Factor V was found [15]. This mutation increases the risk for thromboembolism and miscarriage independently of maternal COVID-19. In that case, foetal death by placental thromboembolism could have been triggered by symptomatic COVID-19 only, but an exacerbation due to Leiden Factor V mutation unrelated to the maternal infection seems more likely. In contrast to other descriptions of placental SARS-CoV-2-infections [9, 15–17], there were no severe foetal nor maternal comorbidity in our case. By no confounding comorbidity known, we can assess the histological impact of maternal SARS-CoV-2-infection on the placental barrier.

Most cases of vertical transmission end at the syncytiotrophoblast layer of the villous surface [18]. Klicken oder tippen Sie hier, um Text einzugeben. This was consistent with results of our in situ padlock hybridisation (Fig. 2). Since we could not detect canonical SARS-CoV-2 entry receptors *ACE2* and *TMPRSS2* with validated RNA probes specific down to the level of a single nucleotide, we tested if detection of SARS-CoV-2 probes is due to necrosis or normal at 35th week of gestation by referring to single cell RNA sequencing (scRNA-seq) data published by Pique-Regi et al. [19]. Single cell and nuclei RNA sequencing dataKlicken oder tippen Sie hier, um Text einzugeben. show missing placental expression of viral entry receptors *ACE2* and *TMPRSS2* and thus unlikely co-expression of *ACE2* and *TMPRSS2* in the placental tissues. Based on a cohort of 252 first and third trimester whole tissue lysate placental samples from our previous studies [20], we can correlate scarce trophoblastic *ACE2* receptor expression in both first and third trimester with a slight increase over gestation. Pique-Regi et al. could find an expression of ACE2 above background in microarray data, where *ACE2* expression was at a negligible level. This may explain a slightly higher risk of placental SARS-CoV-2 viral later in pregnancy.

Maternal vascular malperfusion has been reported in third trimester placentas from the very beginning of the COVID-19 pandemic [21]. SARS-CoV-2 is known to interact with endothelial cells and can lead to the formation of microthrombi [22]. The intervillous space in the placenta is like a large maternal vascular space and in case of maternal viraemia, this space is flooded with viral particles that can be found within the perivillous fibrin deposits. However, the placental barrier is only rarely overcome and the fetus subsequently infected, mostly in cases of severe maternal COVID-19 [16, 23]. Foetal demise by other causes, especially common in first trimester, causes the placental barrier to permeabilise and thus facilitate virus entry through damaged tissue. The case report by Shende et al. described *hydrops foetalis* associated with an asymptomatic SARS-CoV-2-infection in first trimester [17]. In this case, maternal SARS-CoV-2 infection may occur coincidental in combination with other pathologies causing foetal death.

A default vertical transmission and transplacental foetal infection thus seems very unlikely when an intact placental barrier is present. More so, we suggest that placental inflammation only occurs after microthrombotic events induced by maternal COVID-19 damage the placental barrier and thus enable infection. The disease is well known to be thrombogenic and to be causing coagulation abnormalities [24] as well as vascular endothelialitis [25]. We suggest that viral particles are derived by intervillous maternal blood into their perivillous position found in thrombotic material that is causing necrosis of the trophoblastic barrier. This hypothesis is based on scarce to no viral entry receptor detection with massive viral particle detection in areas where trophoblast marker cytokeratin-7 is lacking. A vertical transmission may occur or not, depending on the severity of damage to the placental barrier via COVID-19-related malperfusion and thrombosis in the microvascular placental bed. This is supported by data that foetal infection occurs in severe maternal COVID-19 cases but not in mild disease courses [16]. Most studies looking at possible pathomechanisms have focused on immunological events [12].

In the case presented, the placental weight at 35 + 6 indicates a stagnation of growth since the first visit at 34 + 3. Based on these pathological placental findings, we suggest that maternal COVID-19 leads to local microthrombotic events that may finally result in placental insufficiency. Further research, including larger clinical studies, needs to be conducted to elucidate possible causes for adverse outcomes.

### Electronic supplementary material

Below is the link to the electronic supplementary material.

Supplementary Material 1

Supplementary Material 2

Supplementary Material 3

## Data Availability

The data underlying this article will be shared on reasonable request to the corresponding author.

## Abbreviations

PI: Pulsatility index
IL-8: Interleukin 8
Ct: Cycle threshold
IU: International units
CRP: C-reactive protein
GGT: Gamma-glutamyl transferase
LDH: Lactate dehydrogenase
