# Supplementary material for: Maternal COVID-19 causing intrauterine foetal demise with microthrombotic placental insufficiency: a case report
Source: BMC Pregnancy Childbirth. 2023 Sep 9;23:653. doi: 10.1186/s12884-023-05942-6 (PMC10492311; doi:10.1186/s12884-023-05942-6)
Supplement: Supplementary file 2 — Supplementary Material 2 [file 12884_2023_5942_MOESM2_ESM.docx]

**Data Supplement**

**Figure S1**


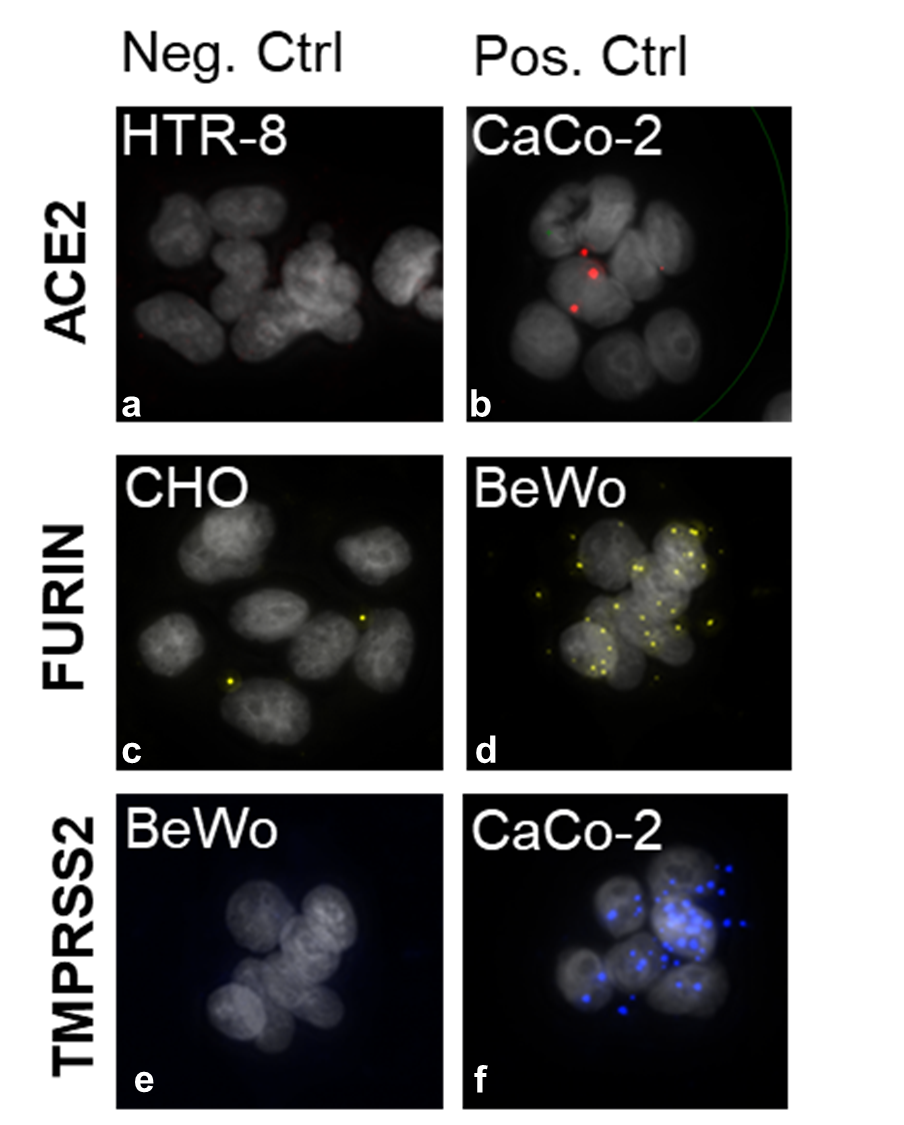


**Validation of *in situ* padlock probes for *ACE2, FURIN, TMPRSS2*.** Cell lines were first tested for expression of *ACE2, FURIN, TMPRSS2*. Cells not expressing the target were used as negative control, those expressing the target for *in situ* padlock probes were used as validation: ACE2 (**a,b**), FURIN (**c, d**), and TMPRSS2 (**e, f**)

*Maternal COVID-19 causing intrauterine foetal demise due to microthrombotic placental insufficiency: a case report.*

*2023*
