# Supplementary material for: Maternal COVID-19 causing intrauterine foetal demise with microthrombotic placental insufficiency: a case report
Source: BMC Pregnancy Childbirth. 2023 Sep 9;23:653. doi: 10.1186/s12884-023-05942-6 (PMC10492311; doi:10.1186/s12884-023-05942-6)
Supplement: Supplementary file 3 — Supplementary Material 3 [file 12884_2023_5942_MOESM3_ESM.docx]

**Maternal COVID-19 causing intrauterine foetal demise due to microthrombotic placental insufficiency: a case report.**

# Material and Methods

## *In situ* Hybridisation

*In situ* hybridisation with padlock probe technology was used to localize SARS-CoV-2 RNA as well as *ACE2*, *FURIN*, *TMPRSS2*, and *ACTB* mRNA transcripts. *In situ* padlock probe-based mRNA detection procedures were performed as described previously (El-Heliebi *et al.*, 2017). In short, the target-mRNA was reverse transcribed and linear oligonucleotides (*i.e.* padlock probes) bound with their 5´- and 3´-parts to the complementary cDNA sequence forming a circular DNA structure. After ligation, this circular DNA structure was amplified forming micron sized DNA structures, which was targeted by fluorescent probes (Weibrecht *et al.*, 2013). Reference sequences were retrieved from the National Centre for Biotechnology Information (GenBank accession numbers NC_045512.2 (SARS-CoV-2), NM_021804.3 (ACE2), NM_001289823.1 (FURIN), NM_001135099.1 (*TMPRSS2*), and NM_001101.5 (*ACTB*). Padlock probes were designed with the help of an open-source Python software package (<https://github.com/Moldia/multi_padlock_design>) as described by Gyllborg et al. (Gyllborg *et al.*, 2020). In addition to the target-specific ends, padlock probe backbones contained a unique barcode for each RNA and shared a common “anchor” sequence for dual-colour staining. For oligonucleotide sequences used see the Data Supplement (**Table S1**).

The *in situ* hybridisation probes were validated on positive and negative controls of cell lines and validated via qPCR for the RNA expression of the targets (Data Supplement, **Figure S1**).

SARS-CoV-2 Hybridisation
Padlock probes for ssRNA of SARS-CoV2 were designed binding to virus regions showing no genetic identity to similar viruses such as MERS-CoV or other SARS-CoV strains; however, such regions are relatively conserved across SARS-CoV2 mutants (Chan *et al.*, 2020; Lu *et al.*, 2020).
The ssRNA reference sequence of SARS-CoV2 Wuhan-Hu-1 isolate was retrieved from the National Center for Biotechnology Information (NCBI, GenBank accession number NC_045512.2). Padlock probes were designed to bind to the S, P13 and N regions. To validate the location of the probes across different SARS-CoV2 mutants, padlock probes were compared to a SARS-COV-2 variant (GenBank accession number NC_045512.2) using the Main Workbench software (CLC Bio Workbench Version 7.6, Qiagen; Venlo, Netherlands).
Primer and padlock probes for ssRNA SARS-CoV2 detection were designed in accordance with the published 2019-Novel Coronavirus (2019-nCoV) Real-time rRT-PCR Panel (https://www.cdc.gov/coronavirus/2019-ncov/lab/rt-pcr-panel-primer-probes.html) and validated on positive (artificially infected tissue) and negative controls (non-infected tissue) as well as via qPCR for the RNA expression of the targets.

## Immunofluorescence staining

The immunofluorescence staining was incorporated into the *in situ* hybridisation protocol and used on the same section. Formalin fixed and paraffin embedded (FFPE) placenta sections were deparaffinised, antigen retrieval was performed using pepsin, and sections were subsequently labelled with primary antibody anti-cytokeratin-7 (1:1000; clone: OV-TL (1352 P); rabbit; Thermo Scientific) and secondary anti-rabbit Alexa Fluor 555 antibody (1:200, Invitrogen, Carlsbad; CA, USA) for 30 min each. After three washing steps in PBS, nuclei were stained with DAPI by mounting sections with Vectashield (Vector Laboratories, Burlingame, CA, USA).

## Laboratory Diagnostics for SARS-CoV-2

RT-qPCR was performed using a Cepheid GeneXpert (Cepheid, Austria) with Xpert Xpress SARS-CoV-2 Assay cartridge technology (Cepheid, Austria).
